# Risk factors for severe gastrointestinal toxicity in patients receiving palliative radiotherapy for metastatic bone tumors: association with the use of molecular-targeted agents

**DOI:** 10.1093/jrr/rraa035

**Published:** 2020-06-22

**Authors:** Yuji Murakami, Masahiro Kenjo, Kazuki Ishikawa, Toru Sakayauchi, Satoshi Itasaka, Yoshiharu Negoro, Keiichi Jingu, Yasumasa Nishimura, Yasushi Nagata, Kazuhiko Ogawa

**Affiliations:** 1 Department of Radiation Oncology, Hiroshima University Hospital, Hiroshima, Japan; 2 Hiroshima High-Precision Radiotherapy Cancer Center, Hiroshima, Japan; 3 Department of Radiation Oncology, Kindai University Faculty of Medicine, Osaka-Sayama, Japan; 4 Department of Radiation Oncology, Osaki Citizen Hospital, Osaki, Japan; 5 Department of Radiation Oncology, Kurashiki Central Hospital, Kurashiki, Japan; 6 Department of Radiation Oncology, Japanese Red Cross Wakayama Medical Center, Wakayama, Japan; 7 Department of Radiation Oncology, Tohoku University Graduate School of Medicine, Sendai, Japan; 8 Department of Radiation Oncology, Osaka University Graduate School of Medicine, Suita, Osaka, Japan

**Keywords:** palliative radiotherapy, bone metastasis, gastrointestinal toxicity, molecularly targeted therapy, vascular endothelial growth factor-targeted agent

## Abstract

This study aimed to investigate whether the use of molecular-targeted agents could affect gastrointestinal (GI) toxicity in palliative radiotherapy (RT) for metastatic bone tumors in the abdominopelvic region. We collected data of patients who received palliative RT for bone metastases in the abdominopelvic region between 2013 and 2014 from six institutions. Data of 395 patients were collected and184 patients received molecularly targeted therapy, of whom 80 received vascular endothelial growth factor (VEGF)-targeted agents. For 556 lesions, 410 sessions of irradiation were undergone. GI toxicity of ≥G3 was observed in 3.8% of patients. The incidence rates of ≥G3 GI toxicity in patients without targeted agents use, in those using VEGF-targeted agents and in those using non-VEGF-targeted agents were 3.8, 7.5 and 1.0%, respectively. Regarding risk factors of the occurrence of ≥G3 GI toxicity, univariate analysis in all patients showed that a history of abdominopelvic surgery was a significant risk factor (*P* = 0.01), and the use of VEGF-targeted agents showed a trend of high incidence (*P* = 0.06). In patients using VEGF-targeted agents, both univariate and multivariate analysis showed that combined anticoagulant use (*P* = 0.03 and 0.01) and agent use between 1 week before and after RT (*P* = 0.046 and 0.03) were significant risk factors. In conclusion, the history of abdominopelvic surgery was associated with ≥G3 GI toxicity and the use of VEGF-targeted agents showed a trend for high incidence. When using VEGF-targeted agents, caution should be exercised in the combined use of anticoagulants and in the agent use between 1 week before and after RT.

## INTRODUCTION

Molecular-targeted agents are drugs that block the growth of cancer cells by interfering with specific targeted molecules and they play an essential role in current cancer treatment. As molecular-targeted agents act specifically on cancer cells at the beginning of development, they were expected to reduce toxicities compared with conventional cytotoxic chemotherapeutic agents. However, severe toxicities have recently emerged, such as interstitial lung disease due to epidermal growth factor receptor-tyrosine kinase inhibitors [1] and bleeding or perforation events due to vascular endothelial growth factor (VEGF)-targeted agents [2].

Patients with bone metastases often receive palliative radiotherapy (RT) for pain relief, prevention of spinal cord paralysis or prevention of fracture. In clinical practice, we often encounter the situation where patients need to receive palliative RT for bone metastases at the time as using molecular-targeted agents. However, the safety of the combination of palliative RT and molecularly targeted therapy is not well known.

In this study, we investigated whether the use of molecular-targeted agents could affect gastrointestinal (GI) toxicity in palliative RT for metastatic bone tumors in the abdominopelvic region, where the gastrointestinal tract was included in the radiation beam pathway.

## MATERIALS AND METHODS

### Inclusion criteria

This study included patients who started RT from 1 January 2013 to 31 December 2014 and who met the following inclusion criteria. (i) Diagnosed with metastatic bone tumors. (ii) Received palliative RT for bone lesions. (iii) The irradiation site included a lower thoracic spine, lumbar spine or pelvic bone where the GI tract was in the irradiation path. (iv) Irradiated with a total dose of 8 Gy or more. (v) No history of abdominal or pelvic RT. (vi) Identified the use or not use of molecular-targeted agents before, during or within 4 weeks after RT. (vii) A follow-up period of ≥8 weeks after the start of RT. (Patients who died 1–8 weeks after the start of RT were included). (viii) No findings of cancerous peritonitis at the beginning of RT.

### Data collection

Data were collected from six institutions that took part in the Japanese Radiation Oncology Study Group (JROSG), Working Subgroup of Gastrointestinal Cancers for this retrospective study. The data collected included information on patient characteristics, primary tumor, bone metastases, RT, the use and details of molecular-targeted agents, GI toxicity and outcomes.

### GI toxicity

We categorized GI toxicities as perforation, bleeding, obstruction (including ileus), diarrhea, inflammation and vomiting. Toxicity was graded according to the Common Terminology Criteria for Adverse Events version 4.0.

### Statistics

We used Fisher’s exact test for univariate analysis and logistic regression for multivariate analysis. For comparison between the three groups, we used the Steel–Dwass method as a multiple comparison method. A *P*-value < 0.05 was considered statistically significant. We performed a statistical analysis using the BellCurve for Excel (Social Survey Research Information Co., Ltd).

### Ethics

The ethical committees of all participating institutions approved this retrospective study.

## RESULTS

### Patient and tumor demographics

The data of 395 patients were collected. Table 1 shows the patient and tumor demographics. There were 160 females and 235 males. The median age was 68 years. Patients with a performance status score of 0–1, 2–4 and unknown were 206, 152 and 37, respectively. The major primary site was lung in 120 patients. There were 556 metastatic bone lesions: 289 spinal legions and 267 pelvic bone legions.

**Table 1:** Patient and tumor demographics

		*n*
Gender	Female/male	160/235
Age (years)	Median (range)	68 (22–92)
Performance status	0–1/2–4/Unknown	206/152/37
Anticoagulant use	Yes/no/unknown	39/353/3
Diabetes mellitus	Yes/no/unknown	43/349/3
Abdominopelvic surgery	Yes/no/unknown	141/245/9
Primary cancer site	Lung	120
	Breast	42
	Colorectal	35
	Prostate	32
	Liver	33
	Kidney	20
	Stomach	17
	Others	96
Bone metastatic site	Spine	289
	Pelvic bone	267

### RT

For 556 metastatic lesions, 410 sessions of irradiation were undergone. Multiple sites (2–4 sites) were irradiated in 116 patients simultaneously or at different times. There were various RT regimens (Table 2), and major regimens were a dose of 30 Gy in 10 fractions (196 sessions, 47.8%) and 20 Gy in 5 fractions (65 sessions, 15.9%).

**Table 2:** Radiotherapy regimens

Dose/fraction (fr)	*n*
8 Gy/1 fr	20
10–19 Gy/3–7 frs	4
20–29 Gy/3–10 frs	107
30–39 Gy/10–15 frs	249
40–49 Gy/16–25 frs	21
50–60 Gy/25–30 frs	9

### Molecularly targeted therapy

A total of 184 (46.6%) patients received molecularly targeted therapy. A single agent was administered in 134 patients, and multiple agents were administered in 50 patients simultaneously or at different times. There were 20 molecular-targeted agents administered, 7 VEGF-targeted agents and 13 non-VEGF-targeted agents (Table 3). VEGF-targeted agents were used in 80 patients. Among them, 47 received VEGF-targeted agents only, and 33 received a combination of VEGF-targeted agents and non-VEGF-targeted agents. Major VEGF-targeted agents used in this study were bevacizumab, sorafenib and sunitinib. Only non-VEGF-targeted agents were used in 104 patients. Major non-VEGF-targeted agents used in this study were denosumab, gefitinib and erlotinib. Patients who received targeted agents between 1 week before and after RT were 21 of 80 patients using VEGF-targeted agents and 49 of 104 patients using non-VEGF-targeted agents only.

**Table 3:** Molecular-targeted agents used in this study

		*n*
VEGF-targeted	Bevacizumab	39
	Sorafenib	24
	Sunitinib	13
	Axitinib	9
	Pazopanib	3
	Regorafenib	2
	Ramucirumab	1
Non-VEGF targeted	Denosumab	49
	Gefitinib	20
	Erlotinib	20
	Trastuzumab	9
	Cetuximab	7
	Everolimus	7
	Crizotinib	5
	Afatinib	4
	Panitumumab	4
	Bortezomib	2
	Temsirolimus	2
	Palbociclib	1
	Lapatinib	1

**Table 4:** Gastrointestinal toxicity

	Grade 2		Grade 3		Grade 4		Grade 5		≥Grade 3	
Obstruction	2	(0.5%)	2	(0.5%)	3	(0.8%)	1	(0.3%)	6	(1.5%)
Bleeding	3	(0.8%)	4	(1.0%)	0		0		4	(1.0%)
Perforation	2	(0.5%)	0		1	(0.3%)	2	(0.5%)	3	(0.8%)
Diarrhea	3	(0.8%)	1	(0.3%)	0		0		1	(0.3%)
Inflammation	4	(1.0%)	0		0		1	(0.3%)	1	(0.3%)
Vomiting	1	(0.3%)	0		0		0		0	
Total	15	(3.8%)	7	(1.8%)	4	(1.0%)	4	(1.0%)	15	(3.8%)

### GI toxicity

#### Toxicity in all patients

The median follow-up duration was 171 (8–1798) days. Thirty (7.6%) patients had G2 or worse GI toxicity and 15 (3.8%) patients had G3 or worse (≥G3) GI toxicity. The median onset of ≥G3 GI toxicity after RT was 131 (1–980) days. The incidence rates of ≥G3 obstruction, bleeding, perforation, diarrhea, inflammation and vomiting were 1.5 (6 patients), 1.0 (4 patients), 0.8 (3 patients), 0.3 (1 patient), 0.3 (1 patient) and 0%, respectively. The incidence rates of life-threatening G4 or G5 events were 1.0 (4 patients), 0, 0.8 (3 patients), 0, 0.3 (1 patient) and 0%, respectively (see Table 4).

#### ≥G3 GI toxicity according to the use of targeted agents

The incidence rates of ≥G3 GI toxicity in patients who did not use the targeted agents, those who used only non-VEGF-targeted agents and those who used VEGF-targeted agents were 3.8 (8 of 211 patients), 1.0 (1 of 104 patients) and 7.5% (6 of 80 patients), respectively (Table 5). The multiple comparisons did not show a significant difference between the three groups. However, there was a trend for high incidence (*P* = 0.055) in patients using VEGF-targeted agents compared with those using non-VEGF targeted agents. Of the six patients using VEGF-targeted agents, two received bevacizumab alone, two received bevacizumab and erlotinib, one received bevacizumab and denosumab and one received sunitinib alone. The patient using non-VEGF-targeted agents received gefitinib alone.

**Table 5:** Use of targeted agents (TA) and ≥Grade 3 toxicity

	No TA use		Non-VEGF-TA		VEGF-TA	
*n*	211		104		80	
Grade 3	4	(1.9%)	0		3	(3.8%)
Grade 4	2	(1.0%)	1	(1.0%)	1	(1.3%)
Grade 5	2	(1.0%)	0		2	(2.5%)
≥Grade 3 toxicity	8	(3.8%)	1	(1.0%)	6	(7.5%)
Obstruction	3	(1.4%)	1	(1.0%)	2	(2.5%)
Bleeding	2	(1.0%)	0		2	(2.5%)
Perforation	2	(1.0%)	0		1	(1.3%)
Diarrhea	1	(0.5%)	0		0	
Inflammation	0		0		1	(1.3%)
Vomiting	0		0		0	

Table 6 shows the analysis results of significant risk factors for the occurrence of ≥G3 GI toxicity. Univariate analysis in all patients showed that the history of abdominopelvic surgery was a significant risk factor (*P* = 0.01) and the use of VEGF-targeted agents showed a trend of high incidence (*P* = 0.06). In patients using VEGF-targeted agents, both univariate and multivariate analysis showed that combined anticoagulant use (*P* = 0.03 and 0.01, respectively) and agent use between 1 week before and after RT (*P* = 0.046 and 0.03, respectively) were significant risk factors.

## DISCUSSION

Bone is the third most common metastatic organ, following the lungs and liver [3]. Bone pain and spinal cord compression are critical complications and cause a significant deterioration in the patient’s quality of life. External beam RT is a popular, useful tool to relieve bone pain and improve or prevent spinal cord compression. In several randomized trials for palliative RT schedules for bone metastases, the occurrence of severe GI toxicity was reported to be relatively rare [4–7]. Foro Arnalot *et al*. reported the results of a randomized clinical trial comparing two irradiation schedules of a single 8 Gy fraction and 30 Gy in 10 fractions. GI toxicity was infrequent, 2% in both groups, and no G3 or G4 toxicity was observed [4]. Hartsell *et al*. reported that G3 GI toxicity was observed in 0.7% of patients receiving 8 Gy in a single fraction, and 1.4% of patients receiving 30 Gy in 10 fractions [5]. In our study, the incidence rate of ≥G3 GI toxicity was slightly higher: 3.8% in all patients. The possible reasons are that the irradiation sites of bone metastases were limited to the abdominopelvic area and that GI toxicities might include not only the toxicity of the treatment but also events due to tumor progression, tumor response or deterioration in patients’ general condition.

Bowel obstruction is a well-known toxicity after abdominopelvic surgery. Small bowel obstruction associated with postoperative adhesions occurs in ~14% of patients [8]. There is little evidence that palliative RT increases the risk of bowel obstruction. However, in this study, bowel obstruction was the most frequent toxicity, and a history of abdominopelvic surgery was a significant factor for ≥G3 GI toxicity. Moreover, the incidence of life-threatening events was relatively high. Therefore, bowel obstruction should be monitored closely in patients with a history of abdominopelvic surgery.

**Table 6:** Analysis of significant risk factors for the occurrence of GI toxicity

		All patients			No TA^a^ use			Non-VEGF-TA use			VEGF-TA use		
Univariate analysis		OR^b^	95% CI^c^	*P*	OR	95% CI	*P*	OR	95%CI	*P*	OR	95% CI	*P*
Gender	Female vs male	0.42	0.61–4.81	0.22	0.55	0.13–2.26	0.31	0.40	0.02–9.96	0.55	0.48	0.09–2.56	0.35
Age (years)	>68 vs ≤68	1.03	0.37–2.89	0.58	0.39	0.08–1.96	0.21	1.76	0.07–44.4	0.63	3.81	0.42–34.2	0.19
Performance status	0-1 vs 2-4	0.39	0.11–1.46	0.11	0.34	0.07–1.75	0.25	0.65	0.03–16.4	0.67	0.37	0.04–3.45	0.34
Anticoagulant use	Yes vs no	0.42	0.11–1.57	0.54	1.60	0.09–29.0	0.48	0.38	0.01–9.92	0.88	0.12	0.02–0.70	0.03
Diabetes mellitus	Yes vs no	1.89	0.24–16.6	0.16	1.23	0.07–22.5	0.50	0.42	0.02–10.9	0.86	3.12	0.17–58.5	0.30
Abdominopelvic surgery	Yes vs no	0.22	0.07–0.71	0.01	0.15	0.03–0.75	0.02	1.27	0.05–32.2	0.69	0.29	0.03–2.69	0.25
Total irradiation dose (Gy)	>30 vs ≤30	0.49	0.16–1.48	0.17	0.53	0.10–2.76	0.19	0.08	0.00–2.08	0.21	1.38	0.15–12.7	0.25
Number of irradiation site	1 vs ≥2	1.21	0.40–3.63	0.46	1.61	0.37–6.98	0.38	0.67	0.03-16.9	0.32	0.42	0.05–3.77	0.41
TA use	Yes vs no	1.00	0.35–2.80	0.60	-	-	-	-	-	-	-	-	-
VEGF-TA use	Yes vs no	2.76	0.95–7.99	0.06	-	-	-	-	-	-	-	-	-
Agent use between 1 week before and after RT	Yes vs no	-	-	-	-	-	-	2.78	0.11–69.7	0.47.	0.16	0.03–0.95	0.046
Multivariate analysis											OR	95% CI	*P*
Anticoagulant use	Yes vs no										0.05	0.01–0.54	0.01
Agent use between 1 week before and after RT	Yes vs no										0.07	0.01–0.74	0.03

^a^TA = targeted agent; ^b^OR = odds ratio; ^c^CI = confidence interval.

VEGF-targeted agents are known as drugs that can cause GI perforation and bleeding. The incidence rate of GI perforation is reported to be between 0.9 and 1.3%. Bevacizumab is the most frequently reported agent, but all other VEGF-targeted agents used in this study have reports of GI perforation, including case reports [2, 9–11]. The incidence rate of GI bleeding is reported to be between 2.4 and 3.4% [12–14]. The detailed mechanism of these toxicities due to the drug itself is not well known. It is speculated that the causes are impairment in the healing process of intestinal mucosal damage by inflammation or therapeutic interventions [15], ischemia due to angiogenesis inhibition or ischemia due to arterial thrombi [16]. Regarding the combination of RT and VEGF-targeted agents, a radiosensitizing effect of VEGF pathway inhibition has also been described in several preclinical studies [17–21]. It is reported that the combination of high-dose RT for intra-abdominopelvic lesions and VEGF-targeted agents increases the risk of severe GI toxicity [22–24]. Several case reports showed the occurrence of severe GI perforation in patients who received a combination of palliative RT and VEGF-targeted agents [25, 26]. In this study, patients using VEGF-targeted agents showed a trend for high incidence of ≥G3 GI toxicity (*P* = 0.06). Even when palliative RT is performed, we should be aware that VEGF-targeted agents could cause severe GI toxicity.

The use of anticoagulants was a significant risk factor in patients using VEGF-targeted agents in this study. Anticoagulants have been reported to trigger GI bleeding [27]. Therefore, special attention should be paid to severe GI bleeding in patients receiving a combination of anticoagulants, VEGF-targeted agents and RT. Another significant risk factor in patients using VEGF-targeted agents was the use of targeted agents between 1 week before and after RT. The optimal schedule of combined treatment with RT and VEGF-targeted agents is unclear. The average half-life listed in the package inserts is ~11–13 days for bevacizumab, 25.5 h for sorafenib, and 49.5 h for sunitinib. From the results of this study, we recommend avoiding the use of VEGF-targeted agents for at least 1 week before and after RT, if possible. However, bevacizumab has a longer half-life, so it may be better to have a drug-free period as long as possible before and after RT.

We recognize the limitations of this study given its retrospective nature, the small number of patients, and a lack of evaluation of the dose-fractionation factor, irradiation field and the use of cytotoxic chemotherapy. Considering the median onset of severe toxicity (131 days), the follow-up period (median 171 days) was not enough. This may be due to the short life expectancy of patients with bone metastases, or due to transfer to palliative care facilities. Moreover, many targeted agents have been developed even after this research period, and the toxicity profile of the combination of new agents and RT must be continuously updated.

In conclusion, a history of abdominopelvic surgery was associated with ≥G3 GI toxicity and the use of VEGF-targeted agents showed a trend for high incidence. When using VEGF-targeted agents, caution should be taken with the combined use of anticoagulants and the use of agents between 1 week before and after RT.
